# A prospective study of dietary selenium intake and risk of type 2 diabetes

**DOI:** 10.1186/1471-2458-10-564

**Published:** 2010-09-21

**Authors:** Saverio Stranges, Sabina Sieri, Marco Vinceti, Sara Grioni, Eliseo Guallar, Martin Laclaustra, Paola Muti, Franco Berrino, Vittorio Krogh

**Affiliations:** 1 Health Sciences Research Institute, University of Warwick Medical School, Coventry, UK; 2 Nutritional Epidemiology Unit, Fondazione IRCCS Istituto Nazionale dei Tumori, Milan, Italy; 3 Department of Public Health Sciences, University of Modena and Reggio Emilia, Modena, Italy; 4 Departments of Epidemiology and Medicine, and Welch Center for Prevention, Epidemiology and Clinical Research, Johns Hopkins Bloomberg School of Public Health, Baltimore, MD, USA; 5 Department of Cardiovascular Epidemiology and Population Genetics, National Center for Cardiovascular Research (CNIC), Madrid, Spain; 6 Epidemiology and Prevention Unit, "Regina Elena" Italian National Cancer Institute, Rome, Italy; 7 Etiological and Preventive Epidemiology Unit, Fondazione IRCCS Istituto Nazionale dei Tumori, Milan, Italy

## Abstract

**Background:**

Growing evidence raises concern about possible associations of high selenium exposure with diabetes in selenium-replete populations such as the US. In countries with lower selenium status, such as Italy, there is little epidemiological evidence on the association between selenium and diabetes. This study examined the prospective association between dietary selenium intake and risk of type 2 diabetes.

**Methods:**

The ORDET cohort study comprised a large sample of women from Northern Italy (n = 7,182). Incident type 2 diabetes was defined as a self-report of a physician diagnosis, use of antidiabetic medication, or a hospitalization discharge. Dietary selenium intake was measured by a semi-quantitative food-frequency questionnaire at the baseline examination (1987-1992). Participants were divided in quintiles based on their baseline dietary selenium intake.

**Results:**

Average selenium intake at baseline was 55.7 μg/day. After a median follow-up of 16 years, 253 women developed diabetes. In multivariate logistic regression analyses, the odds ratio for diabetes comparing the highest to the lowest quintile of selenium intake was 2.39, (95% CI: 1.32, 4.32; *P *for linear trend = 0.005). The odds ratio for diabetes associated with a 10 μg/d increase in selenium intake was 1.29 (95% CI: 1.10, 1.52).

**Conclusions:**

In this population, increased dietary selenium intake was associated with an increased risk of type 2 diabetes. These findings raise additional concerns about the association of selenium intake above the Recommended Dietary Allowance (55 μg/day) with diabetes risk.

## Background

Selenium is a key component of a number of selenoproteins involved in essential enzymatic functions, such as redox homeostasis, thyroid hormone metabolism, immunity and reproduction [1]. Because of antioxidant properties of selenoproteins, and because selenate mimics insulin activity in experimental models [2,3], selenium was expected to prevent type 2 diabetes and cardiovascular disease (CVD) [4]. However, recent findings from observational studies and randomised clinical trials have raised concerns with respect to possible adverse cardio-metabolic effects of high selenium exposure, at least in well-nourished populations. Specifically, several unrelated studies from the US indicate that high selenium status or selenium supplementation is associated with an increased diabetes risk [5-8]. Furthermore, recent evidence from several populations indicates that high selenium exposure may also be associated with an adverse lipid profile [9-12] and hypertension [13], raising additional concerns about metabolic toxicity of high selenium exposure and prolonged use of selenium supplements [14].

Dietary intake of selenium varies considerably between countries and regions largely due to the variability of the selenium content of soil and hence of plant foods and animal forage [15]. Current recommendations on selenium intake are based on optimizing the activity of plasma glutathione peroxidases, which are maximized at intakes as high as 55 μg/day [16]. In the US, selenium intake ranges from 60-220 μg/day [8,12,13,15] and it is unclear if there are health benefits of increased selenium intake above the Recommended Dietary Allowance (RDA), or if metabolic toxicity might occur at these levels. Selenium intakes in Europe are lower than in the US, but with large between-country variability ranging from adequate or marginally adequate (Western and Central Europe: 30-90 μg/day) to low or deficient intake (Eastern European countries: 7-30 μg/day) [15].

There is little epidemiological evidence on the association of selenium with diabetes among European populations [17,18]. The objective of the present study was thus to examine the association of dietary selenium intake with risk of incident type 2 diabetes in the ORDET cohort study, a large sample of women from Northern Italy [19].

## Methods

### Study population

The ORDET study (HORmones and Diet in the ETiology of Breast Cancer) is an ongoing prospective follow-up study of 10,786 women residents of Varese province in Northern Italy [19]. Between June 1987 and June 1992, healthy women 34 to 70 years of age were asked to join the study by coming to the recruitment center. Participants were recruited at public meetings, through radio, television and newspaper advertising, and at breast cancer prevention/early diagnosis units. Women with a history of cancer, bilateral ovariectomy, or liver disease, and women receiving hormone therapy in the three months prior to recruitment to the study were not eligible for the study. We excluded women who did not fill in the lifestyle questionnaire (N = 96), who reported the presence of type 2 diabetes at the baseline assessment (N = 203), who did not compile the food frequency questionnaire because it was not available at the beginning of the study (N = 1,552), or who had missing data in anthropometric variables (N = 54). We further excluded participants in whom the ratio of total energy intake (determined from the food frequency questionnaire) to basal metabolic rate was at either extreme of the distribution (cut-offs 0.5 and 99.5 percentiles) (N = 73), to reduce the impact of implausible extreme values [20], as well as women who died during follow-up from causes other than type 2 diabetes (N = 336). Finally, 1,290 women could not be re-contacted over the follow-up and were further excluded. After all these exclusions, the final cohort comprised 7,182 participants. All participants signed an informed consent form. The Ethical Review Board of the Italian National Cancer Institute of Milan approved the study.

### Baseline measurements

Diet was assessed using a semi-quantitative food-frequency questionnaire designed to capture local dietary habits [19]. Frequency of consumption and average daily consumption of food and beverage items over the previous year were estimated using a 107-item questionnaire. To estimate daily consumption of macronutrients and daily energy intake, these items were linked to the Italian Food Tables for epidemiological studies [21].

We used two sources of data to estimate the selenium content of the food items included in the ORDET food frequency questionnaire. For several food items, we used the nutritional database maintained by the Italian National Institute of Nutrition, which in its 1997 edition reported the average selenium content of several foodstuffs distributed in Italy [22]. Since the selenium composition of some items in the ORDET questionnaire (particularly for cereals and meats) could not be retrieved in this database nor were they available from other national sources, we carried out a specific measurement by selecting samples of these foods distributed in Northern Italy from 1996 through 1998 and determining their average selenium content by hydride-generation atomic absorption spectrometry [23].

A lifestyle questionnaire was also completed by each participant to collect detailed information on reproductive history, alcohol consumption, smoking habits, medical history, occupation, education and other socioeconomic variables. Weight and height were measured at enrolment by trained nurses according to a standardized protocol. Body mass index (BMI) was calculated as weight divided by height squared (kg/m^2^).

### Case ascertainment and verification

Follow-up information was collected through a telephone interview in 2006-2007. The follow-up questionnaire included update information on weight, menopausal status, incident disease and other factors. Follow-up was available for approximately 79.1% of the baseline cohort (mean follow-up: 16 years). The telephone follow-up interviews asked whether participants had received a diagnosis of type 2 diabetes from a physician after the baseline examination or if they were taking medications for treatment of diabetes. In addition to the follow-up questionnaire, information on hospitalizations and medication use during follow-up was obtained by linking regional hospital discharge databases, and prescription drug databases to the ORDET database mainly via social security numbers.

In the present study, incident type 2 diabetes was defined by the presence of at least three of the following conditions: 1) a self-report of a physician diagnosis in the follow-up questionnaire; 2) a self-report of use of insulin or oral hypoglycemic medication in the follow-up questionnaire; 3) evidence of a prescription for insulin or oral hypoglycemic medication by linkage with regional prescription drug database; or 4) a hospital discharge record with the diagnosis of diabetes by linkage with medical discharge records. These criteria complied with the protocol of the InterAct Study, an EU-funded large scale collaboration involving nine European Countries and 500,000 subjects with over 10,000 incident diabetic cases http://www.inter-act.eu/.

### Statistical analysis

The study population was categorized in quintiles of energy-adjusted selenium intake at baseline using the residual method [20]. Odds ratios (OR) for developing type 2 diabetes comparing the highest to the lowest quintile of selenium intake were estimated by logistic regression analysis. We used two levels of adjustment: model 1 (reduced model) was adjusted for age, education and menopausal status; model 2 (fully-adjusted model) was further adjusted for BMI (as a linear term), smoking (never, past, current), alcohol intake (abstainers, ≤ 12 g/day, > 12 g/day), energy intake (not from alcohol), saturated/polyunsaturated fat ratio, animal proteins, total carbohydrates, and body weight change (delta-weight) between the baseline and follow-up examinations. Tests for trend across selenium intake quintiles were derived from likelihood ratio tests comparing models with and without a variable including the median selenium intake at each quintile as a continuous variable. We tested the interaction of selenium intake with BMI categories (BMI ≤ 25 & > 25) and with menopausal status using a likelihood ratio test that compared the model that included the product term and the model that did not include it. We used STATA software (version 10.0; Stata Corp., TX) for statistical analysis.

## Results

Average selenium intake at baseline was 55.7 μg/day. After a median follow-up of 16 years, 253 women developed type 2 diabetes. At baseline, women who developed diabetes over follow-up were on average older, heavier, less educated, had higher dietary intakes of total and animal proteins, consumed less alcohol, and were more likely to be postmenopausal than women who did not develop diabetes. In addition, they had a higher mean dietary intake of selenium (60.9 vs. 56.8 μg/d, *P *< 0.001) (Table 1).

**Table 1:** Baseline characteristics* of participants according to diabetes status at the follow-up examination in the ORDET Study (N = 7,182)

	Diabetic cases	Non-diabetics	*P*
N	253	6,929	
**Selected characteristics**			
Age	51.2	47.1	< 0.001
BMI (kg/m^2^)	29.5 (0.25)	24.8 (0.05)	< 0.001
Current smoker (%)	17.8	19.8	0.43
Education (%) > 8 years	36.0	54.3	< 0.001
Post-menopausal status (%)	50.0	32.9	< 0.001
Total proteins (g/day)	79.8 (1.42)	75.8 (0.27)	0.006
Animal proteins (g/day)	54.5 (1.19)	51.2 (0.23)	0.006
Animal fat	37.6 (0.95)	38.0 (0.18)	0.74
Ratio saturated/polyunsaturated fatty acids	0.32 (0.008)	0.31 (0.001)	0.18
Total carbohydrates (g/day)	215.1 (4.00)	215.4(0.76)	0.96
Starch (g/day)	146.5 (3.13)	145.5(0.60)	0.75
Sugars (g/day)	68.6 (1.67)	69.9(0.32)	0.47
Fibers (g/day)	20.3 (0.07)	19.7 (0.38)	0.12
Alcohol (g/day)	8.2 (0.81)	10.0(0.16)	0.03
Energy (kcal/day)	1789 (30.18)	1788 (5.74)	0.97
Selenium intake (μg/day)	60.9 (1.11)	56.8 (0.212)	< 0.001

* Age-adjusted means (standard error), except where indicated.

Red meat and fish were the two main sources of dietary selenium intake in this population (Table 2). Selenium intake was positively associated with BMI, with total and animal protein intake, and with the ratio of polyunsaturated to saturated fatty acid intake (Table 3). Conversely, intake of total carbohydrates, starch, sugars fibers and alcohol were all inversely associated with dietary selenium intake.

**Table 2:** Food sources of selenium in the ORDET Study

Food items	%
Red meat	21.7
Fish	14.5
White meat	7.0
Processed meat	7.0
Cheese	5.7
Pasta	5.4
Bread	5.2
Eggs	5.1
Fruit	3.8
Tomatoes	3.8
Milk	3.7
Cake	3.6
Offal	2.7
Snacks	1.8
Wine	1.8
Rice	1.0
Yoghurt	0.9
Potatoes	0.8
Leafy vegetable raw	0.8

**Table 3:** Baseline characteristics* of participants according to energy-adjusted quintiles† of dietary selenium intake in the ORDET Study (N = 7,182)

	Quintiles of dietary selenium intake (μg/day)					
	**I**	**II**	**III**	**IV**	**V**	***P *trend**
N	1,437	1,436	1,437	1,436	1,436	
Average Se intake (μg/d)	41.7	50.2	55.7	62.0	75.1	-
Age	47.3	47.1	47.6	47.3	46.9	0.37
BMI (kg/m^2^)	24.2	24.5	24.8	25.3	25.9	< 0.001
Current smoker (%)	19.1	18.5	18.3	21.9	21.0	0.03
Education (%) > 8 years	56.5	53.6	51.5	53.7	52.9	0.09
Post-menopausal status (%)	34.0	35.7	32.8	34.6	31.1	0.08
Total proteins (g/day)	67.1	69.2	72.6	78.9	91.9	< 0.001
Animal proteins (g/day)	39.9	44.7	48.7	55.1	68.1	< 0.001
Animal fat	36.3	36.4	36.5	38.5	42.1	< 0.001
Ratio saturated/polyunsaturated fatty acids	0.28	0.31	0.31	0.32	0.34	< 0.001
Total carbohydrates (g/day)	240.5	216.4	209.0	207.3	203.6	< 0.001
Starch (g/day)	164.0	147.5	141.3	139.6	135.2	< 0.001
Sugars (g/day)	76.5	68.8	67.7	67.7	68.4	< 0.001
Fibers (g/day)	21.2	19.1	18.9	19.1	19.5	< 0.001
Alcohol (g/day)	10.3	10.3	10.4	9.9	8.8	0.001
Energy (kcal/day)	1843	1741	1728	1768	1840	0.59

* Age-adjusted means, except where indicated

† Quintiles of dietary Se intake were adjusted for total energy intake using the residual method [20]

The age, education, and menopausal status adjusted odds ratio for incident type 2 diabetes comparing the highest to the lowest quintile of selenium intake was 2.64 (95% CI: 1.73, 4.01), with evidence of a progressive increase in risk across quintiles (*P*-trend < 0.001) (Table 4). The odds ratio estimates were not considerably altered after additional adjustment for BMI, smoking status, dietary variables and body weight change during follow-up (OR 2.39, 95% CI: 1.32, 4.32).

**Table 4:** Odds Ratios (95% Confidence Intervals) of incident type 2 diabetes by quintiles* of dietary selenium intake in the ORDET Study

	Quintiles of dietary selenium intake (μg/day)					
	**I**	**II**	**III**	**IV**	**V**	***P *trend**†
N° cases of diabetes	32	42	45	55	79	
Range Se intake (μg/day)	≤ 47.0	47.1-53.0	53.1-58.5	58.6-65.9	> 65.9	
Model 1 (reduced)	1.00	1.31 (0.82-2.09)	1.38 (0.87-2.19)	1.74 (1.12-2.72)	2.64 (1.73-4.01)	< 0.001
Model 2 (fully-adjusted)	1.00	1.42 (0.87-2.34)	1.43 (0.86-2.38)	1.65 (0.98-2.78)	2.39 (1.32-4.32)	0.005

* Quintiles of dietary selenium intake were adjusted for total energy intake using the residual method [20]

Model 1: Adjusted for age, education, menopausal status

Model 2: Adjusted for age, education, menopausal status, BMI, smoking (never, past, current), alcohol intake (abstainers, ≤ 12 g/day, > 12 g/day), energy intake (not from alcohol), saturated/polyunsaturated fatty acids ratio, animal proteins, total carbohydrates, and weight change between the baseline and follow-up examinations

† Test for linear trend performed on median intake for each quintile

When selenium was used as a continuous variable, the odds ratios associated with a 10 μg/d increase in selenium intake were 1.29 (95% CI: 1.17, 1.41) in the reduced model, and 1.29 (95% CI: 1.10, 1.52) in the fully adjusted model. The linearity of the relationship between selenium intake and risk of diabetes was confirmed in spline regression models (not shown). There was no statistical evidence that BMI, menopausal status, smoking and alcohol intake modified the association of selenium intake with diabetes risk (data not shown).

## Discussion

In this prospective study, dietary selenium intake showed a strong and graded association with the risk of type 2 diabetes in a large sample of Italian women. The association was independent of a number of potential confounding factors including socio-demographic, anthropometric, lifestyle and dietary variables. To our knowledge, this is one of the few epidemiological studies to examine the prospective relationship of dietary selenium intake with incident type 2 diabetes in Europe.

Recent findings from observational studies and randomised clinical trials from the US, a selenium-replete population, indicate that high selenium status or selenium supplementation may be associated with an increased risk of type 2 diabetes [5-8]. Data from the Third National Health and Nutrition Examination Survey (NHANES III) [5] and from NHANES 2003-2004 [8] showed significant cross-sectional associations between high serum selenium levels and the prevalence of type 2 diabetes in representative samples of the US population. Furthermore, selenium supplementation (200 μg/d) in the Nutritional Prevention of Cancer (NPC) trial, conducted in the Eastern US, was associated with an increased risk of incident type 2 diabetes compared to placebo (hazard ratio, 1.55, 95% CI: 1.03, 2.33). The increase in risk was largely limited to participants with high baseline selenium levels (hazard ratio of 2.70 in the highest tertile of serum selenium) [6]. Finally, the Selenium and Vitamin E Cancer Prevention Trial (SELECT), conducted among 35,000 North American men aged 50 and older, was prematurely stopped because of lack of efficacy of vitamin E and selenium supplementation (200 μg/d) in cancer prevention and because of a small, though not statistically significant increase in the number of cases of adult onset diabetes in participants taking only selenium (relative risk compared to placebo 1.07, 99% CI: 0.94, 1.22) [7]. In disagreement with these studies, cross-sectional findings from the Health Professionals Follow-up Study showed lower toenail selenium levels among diabetic men (with or without cardiovascular disease) than among healthy control participants [24]. In addition, a longitudinal analysis of US young adults participating in the CARDIA Study showed a lower risk of type 2 diabetes in the highest quintile of toenail selenium compared to the lowest (hazard ratio 0.59, 95% CI: 0.36, 0.97), although the point estimates in the intermediate quintiles were null [25].

With regard to European populations, there is little epidemiological evidence. In the Supplementation with Antioxidant Vitamins and Minerals (SU.VI.MAX) trial, an antioxidant supplement containing 100 μg of selenium, 120 mg of vitamin C, 30 mg of vitamin E, 6 mg of β-carotene, and 20 mg of zinc had no effect on plasma glucose levels after 7.5 years of follow-up, but in this study there was a significant positive association between plasma selenium and glucose levels at both baseline and follow-up [17]. Conversely, a recent report from the EVA (Epidemiology of Vascular Ageing) study in France showed that higher plasma selenium concentrations (1.19-1.97 μmol/L) were associated with a marginally significant decreased risk of dysglycemia (impaired fasting glucose or diabetes) in men over a 9-year follow-up [18]. No association was found in women. It should be noted that in an earlier analysis of the same study, plasma selenium concentrations were positively, though non-significantly, associated with baseline glucose levels in women and with prevalent diabetes in men [26].

Mechanistic evidence that may explain an association between high selenium exposure and increased risk of type 2 diabetes is limited; therefore any such discussion is highly speculative at the present time. Selenium has a narrow therapeutic range and large inter-individual variability in terms of metabolic sensitivity [27,28]. Selenium species such as selenite and selenate may impair insulin responsiveness in rats and induce a catabolic response in muscle with glycogen depletion and increased rates of glycolysis [29]. Moreover, high-selenium diets may stimulate the release of glucagon, promoting hyperglycemia [30], or may induce over-expression of glutathione peroxidase-1 (GPx-1) and other antioxidant selenoproteins resulting in insulin resistance and obesity [31-33]. Likewise in humans, a strongly positive correlation between GPx activity and insulin resistance was found in a group of non-diabetic pregnant women [34]. From a mechanistic point of view, selenium intakes above the level recommended for optimal activity of antioxidant selenoproteins such as glutathione peroxidases (55 μg/day, resulting in serum or plasma concentrations of 70-90 μg/L) [16,35], will result in the non-specific incorporation of selenomethionine replacing methionine in albumin and other proteins [1]. The metabolic pathways involving this extra pool of selenium are not fully understood, and may be responsible for some of the adverse effects of high selenium exposure on glucose metabolism.

The present study may suffer from the inherent limitations associated with the use of self-reported semi-quantitative food-frequency questionnaires, an approach prone to misclassification of the exposure of interest. Indeed, the correlation between dietary selenium intake and selenium biomarkers has been inconsistent across studies, ranging from positive, strong associations [36,37] to weak or null associations [38,39]. A clear limitation of our study is the lack of validation data for food-frequency estimates of selenium intake in our population, which makes it impossible for us to correct selenium and other nutritional covariates for random or systematic measurement error. However, while the use of questionnaire data to estimate selenium intake has raised concerns in settings with high variability in the selenium content of foods and high frequency of use of vitamin and mineral supplements, several factors point to the validity of the selenium intake estimates in the present study. First, the ORDET study compiled a detailed database of the selenium content of local foods, with *ad hoc *measurements of the selenium content of a variety of foods. Second, previous studies have shown little variability of selenium in food contents as well as of selenium status throughout Italy [40], making it easier to estimate selenium intake through questionnaire data. Indeed, the range of dietary selenium intake in the present study is compatible with previous estimates of selenium status, based on biomarkers, from Italian populations [40-42]. Finally, despite the lack of data on the use of dietary supplements containing selenium in the ORDET study, a nationwide survey conducted in Italy in the 90s [43] showed a small percentage of supplement users among women (less than 5%). Thus, it is likely that very few participants were using supplements during the 80s when the ORDET sample was recruited. In our setting, assessing selenium exposure through evaluation of dietary intake may even have advantages over selenium biomarkers, which may be influenced by the intake of other nutrients such as methionine [41], by smoking and other life-style variables [44], drug use [44], and the chemical species of selenium itself [35].

As a further potential limitation of the present study, we cannot rule out confounding effects by unmeasured (e.g. family history of diabetes, physical activity) or unknown factors that may have contributed to our findings. The association between selenium intake and diabetes could be driven by a common dietary factor or by general over-nutrition. For example, red meat, the main source of selenium in our study, has been positively associated with type 2 diabetes in prospective settings among women [45], and contains compounds such as preservatives, additives, and nitrates that might confound the observed associations of selenium intake with diabetes risk.

Our study may have underestimated the incidence of type 2 diabetes, as case identification was based on self-report and record linkage to hospital and prescription databases. It should be noted, however, that case ascertainment of incident diabetes in the follow up of the ORDET study complied with the protocol of the InterAct Study, an EU-funded large scale collaboration involving 350,000 subjects with over 10,000 incident diabetic cases http://www.inter-act.eu/. In addition, given the voluntary nature of the study population, we cannot rule out the potential for a "healthy volunteer' bias, which may have contributed to a low incidence of type 2 diabetes in this study.

Finally, given the prospective design, differential misclassification seems unlikely and non-differential misclassification would result in an underestimation of the association between selenium intake and diabetes risk [46]. Major strengths of the present study are its prospective nature, which limits the possibility of reverse causality, high standardization of data collection, length and high rate of follow-up.

## Conclusions

The use of selenium enriched foods, fertilizers, and supplements has increased markedly in many Western countries in recent years [47,48] because of the perception that selenium can potentially reduce the risk of cancer and other chronic diseases. Hence, from a public health perspective it is essential to ensure that selenium fortification of the food supply and use of selenium supplements do not exacerbate the current diabetes epidemic.

The present study adds to the evidence of an association between high selenium exposure and potential diabetes risk. Future studies are needed to investigate the link between selenium exposure and metabolic effects in more detail across different ranges of exposure, as well as potential underlying mechanisms [14,49].

## Competing interests

The authors declare that they have no competing interests.

## Authors' contributions

SSt, SSi, MV and VK participated in the planning and conception of the research questions and the study design. SSi, SG and VK were responsible for retrieving and analyzing the data. SSt drafted the article, and all authors participated in interpreting the data and critically revising the manuscript for important intellectual content. All authors read and approved the revised manuscript.

## Pre-publication history

The pre-publication history for this paper can be accessed here:

http://www.biomedcentral.com/1471-2458/10/564/prepub
